# Oxido[*N*-(2-oxidobenzyl­idene-κ*O*)leucinato-κ^2^ *N*,*O*](1,10-phenanthroline-κ^2^ *N*,*N*′)vanadium(IV)

**DOI:** 10.1107/S1600536812025391

**Published:** 2012-06-13

**Authors:** Cheng-Yuan Wang, Bu-Qin Jing, Jian-Fang Dong, Lian-Zhi Li

**Affiliations:** a Research Center of Medical Chemistry and Chemical Biology, Chongqing Technology and Business University, Chongqing 400067, People’s Republic of China; b School of Chemistry and Chemical Engineering, Liaocheng University, Shandong 252059, People’s Republic of China

## Abstract

In the title V^IV^ complex, [VO(C_13_H_15_NO_3_)(C_12_H_8_N_2_)], the oxidovanadium cation is *N*,*N*′-chelated by a 1-10-phenanthroline ligand and *N*,*O*,*O*′-chelated by a Schiff base anion in a distorted octa­hedral geometry. Weak inter­molecular C—H⋯O hydrogen bonds occur in the crystal structure which contains solvent-accessible voids of 81 Å^3^.

## Related literature

For the biological and pharmacological properties of vanadium complexes, see: Baran (2003 ▶). For the structures of similar six-coordinate oxidovanadium complexes with amino acid Schiff base ligands, see: Bian *et al.* (2011 ▶); Cao *et al.* (2011 ▶); Xu *et al.* (2005 ▶); Li *et al.* (2006 ▶, 2010 ▶); Lu *et al.* (2011 ▶); Sasmal *et al.* (2007 ▶).
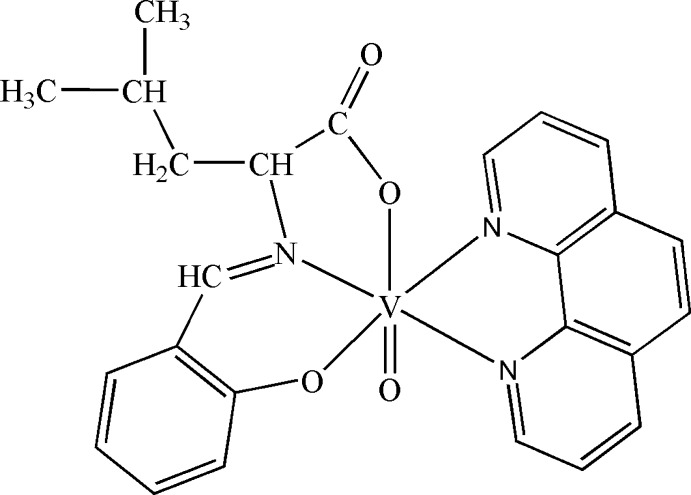


## Experimental

### 

#### Crystal data

[V(C_13_H_15_NO_3_)O(C_12_H_8_N_2_)]
*M* *_r_* = 480.40
Hexagonal, 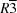

*a* = 33.675 (4) Å
*c* = 10.283 (2) Å
*V* = 10099 (3) Å^3^
*Z* = 18
Mo *K*α radiation
μ = 0.48 mm^−1^
*T* = 298 K
0.23 × 0.11 × 0.08 mm

#### Data collection

Bruker SMART 1000 CCD area-detector diffractometer
Absorption correction: multi-scan (*SADABS*; Bruker, 2001 ▶) *T*_min_ = 0.898, *T*_max_ = 0.963
17437 measured reflections
3962 independent reflections
2020 reflections with *I* > 2σ(*I*)
*R*_int_ = 0.137

#### Refinement

*R*[*F* ^2^ > 2σ(*F* ^2^)] = 0.065
*wR*(*F* ^2^) = 0.128
*S* = 1.00
3962 reflections
298 parameters
H-atom parameters constrained
Δρ_max_ = 0.40 e Å^−3^
Δρ_min_ = −0.38 e Å^−3^

### 

Data collection: *SMART* (Bruker, 2007 ▶); cell refinement: *SAINT* (Bruker, 2007 ▶); data reduction: *SAINT*; program(s) used to solve structure: *SHELXS97* (Sheldrick, 2008 ▶); program(s) used to refine structure: *SHELXL97* (Sheldrick, 2008 ▶); molecular graphics: *SHELXTL* (Sheldrick, 2008 ▶) and *DIAMOND* (Brandenburg, 1999 ▶); software used to prepare material for publication: *SHELXTL*.

## Supplementary Material

Crystal structure: contains datablock(s) global, I. DOI: 10.1107/S1600536812025391/xu5555sup1.cif

Structure factors: contains datablock(s) I. DOI: 10.1107/S1600536812025391/xu5555Isup2.hkl

Additional supplementary materials:  crystallographic information; 3D view; checkCIF report

## Figures and Tables

**Table 1:** Selected bond lengths (Å)

V1—O1	1.989 (3)
V1—O3	1.941 (3)
V1—O4	1.587 (3)
V1—N1	2.042 (3)
V1—N2	2.125 (3)
V1—N3	2.340 (3)

**Table 2:** Hydrogen-bond geometry (Å, °)

*D*—H⋯*A*	*D*—H	H⋯*A*	*D*⋯*A*	*D*—H⋯*A*
C12—H12⋯O4^i^	0.93	2.44	3.311 (6)	156
C24—H24⋯O1^ii^	0.93	2.51	3.224 (6)	134

Symmetry codes: (i) 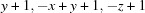; (ii) 
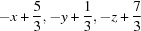
.

**Fig. 1.**
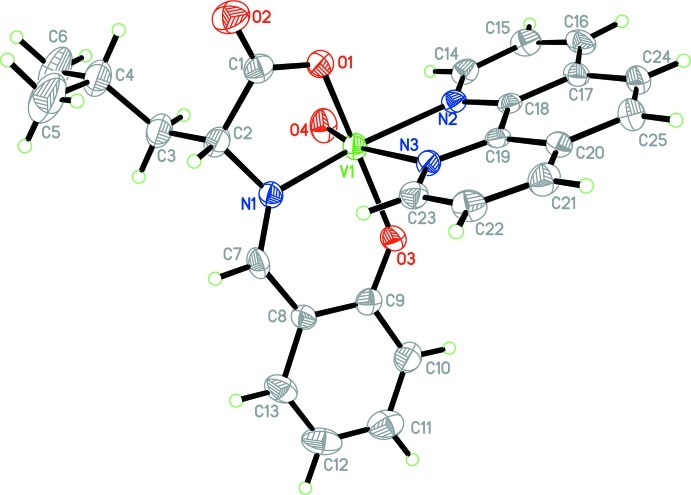
The molecular structure of the title compound, shown 30% probability displacement ellipsoids and the atom-numbering scheme.

## supplementary crystallographic information

### Comment

Vanadium complexes have have been synthesized and characterized continuously due to its biological and pharmacological properties (Baran, 2003). Herein, we report the synthesis and crystal structure of a new oxovanadium(IV) complex with a tridentate Schiff base ligand derived from the condensation of salicylaldehyde and *L*-Leucine, with a 1,10-phenanthroline coligand.

As shown in Fig. 1, the central V(IV) ion is six-coordinated bound to two O atoms and one N atom of the Schiff base ligand, a vanadyl O atom and two N atoms of the 1,10-phenanthroline ligand, forming a distorted octahedral geometry. Selected bond angles and bond distances of the title complex are given in Table 1.

In the molecular structure of the complex, O1, N1, O3 and N2 atoms are in the equatorial plane, O4 and N3 is in the axial position. The V1 ion lies 0.3485 (17) Å above the equatorial plane towards O4. The V1—N3 bond is significantly longer [2.340 (3) Å] (Table 1), similar to the reported vanadium(V) complex (Bian *et al.*, 2011; Cao *et al.*, 2011; Xu *et al.*, 2005; Li *et al.*, 2010; Li *et al.*, 2006;).

In the crystal structure, weak intermolecular C—H···O hydrogen bonds (Table 2) occur.

### Experimental

*L*-Leucine (1 mmol, 131.2 mg) and potassium hydroxide (1 mmol, 56.1 mg) were dissolved in hot methanol (10 ml) with stirring and added successively to a methanol solution (5 ml) of salicylaldehyde (1 mmol, 0.11 ml). The mixture was then stirred at 323 K for 2 h. Subsequently, an aqueous solution (2 ml) of vanadyl sulfate hydrate (1 mmol, 225.4 mg) was added dropwise and stirred for 2 h continuously. Finally, a methanol solution (5 ml) of 1,10-phenanthroline (1 mmol, 198 mg) was added dropwise and stirred for 2 h. Then the resultant solution was filtered and the filtrate was held at room temperature for several days, whereupon yellow blocky crystals suitable for X-ray diffraction were obtained.

### Refinement

All the H atoms were placed in geometrically calculated positions, with C—H = 0.93–0.98 Å and allowed to ride on their respective parent atoms, with *U*_iso_(H) = 1.2*U*_eq_ or 1.5*U*_eq_(C_methyl_).

### Crystal data

**Table:**

[V(C_13_H_15_NO_3_)O(C_12_H_8_N_2_)]	*D*_x_ = 1.422 Mg m^−^^3^
*M**_r_* = 480.40	Mo *K*α radiation, λ = 0.71073 Å
Hexagonal, *R*3	Cell parameters from 3014 reflections
Hall symbol: -R 3	θ = 2.4–28.3°
*a* = 33.675 (4) Å	µ = 0.48 mm^−^^1^
*c* = 10.283 (2) Å	*T* = 298 K
*V* = 10099 (3) Å^3^	Block, yellow
*Z* = 18	0.23 × 0.11 × 0.08 mm
*F*(000) = 4482	

### Data collection

**Table:**

Bruker SMART 1000 CCD area-detector diffractometer	3962 independent reflections
Radiation source: fine-focus sealed tube	2020 reflections with *I* > 2σ(*I*)
Graphite monochromator	*R*_int_ = 0.137
φ and ω scans	θ_max_ = 25.0°, θ_min_ = 2.1°
Absorption correction: multi-scan (*SADABS*; Bruker, 2001)	*h* = −35→40
*T*_min_ = 0.898, *T*_max_ = 0.963	*k* = −39→39
17437 measured reflections	*l* = −12→11

### Refinement

**Table:**

Refinement on *F*^2^	Primary atom site location: structure-invariant direct methods
Least-squares matrix: full	Secondary atom site location: difference Fourier map
*R*[*F*^2^ > 2σ(*F*^2^)] = 0.065	Hydrogen site location: inferred from neighbouring sites
*wR*(*F*^2^) = 0.128	H-atom parameters constrained
*S* = 1.00	*w* = 1/[σ^2^(*F*_o_^2^) + (0.0477*P*)^2^] where *P* = (*F*_o_^2^ + 2*F*_c_^2^)/3
3962 reflections	(Δ/σ)_max_ < 0.001
298 parameters	Δρ_max_ = 0.40 e Å^−^^3^
0 restraints	Δρ_min_ = −0.38 e Å^−^^3^

### Special details

**Table:**

Geometry. All e.s.d.'s (except the e.s.d. in the dihedral angle between two l.s. planes) are estimated using the full covariance matrix. The cell e.s.d.'s are taken into account individually in the estimation of e.s.d.'s in distances, angles and torsion angles; correlations between e.s.d.'s in cell parameters are only used when they are defined by crystal symmetry. An approximate (isotropic) treatment of cell e.s.d.'s is used for estimating e.s.d.'s involving l.s. planes.
Refinement. Refinement of *F*^2^ against ALL reflections. The weighted *R*-factor *wR* and goodness of fit *S* are based on *F*^2^, conventional *R*-factors *R* are based on *F*, with *F* set to zero for negative *F*^2^. The threshold expression of *F*^2^ > σ(*F*^2^) is used only for calculating *R*-factors(gt) *etc*. and is not relevant to the choice of reflections for refinement. *R*-factors based on *F*^2^ are statistically about twice as large as those based on *F*, and *R*- factors based on ALL data will be even larger.

### Fractional atomic coordinates and isotropic or equivalent isotropic displacement parameters (Å^2^)

**Table:**

	*x*	*y*	*z*	*U*_iso_*/*U*_eq_	
V1	0.88840 (2)	0.14374 (2)	0.82377 (7)	0.0405 (3)	
N1	0.93728 (11)	0.12709 (11)	0.7777 (3)	0.0400 (9)	
N2	0.85177 (11)	0.17154 (11)	0.9193 (3)	0.0364 (9)	
N3	0.93599 (11)	0.19076 (10)	0.9904 (3)	0.0344 (9)	
O1	0.87689 (9)	0.09726 (9)	0.9600 (3)	0.0488 (8)	
O2	0.89395 (11)	0.04585 (11)	1.0382 (3)	0.0667 (10)	
O3	0.92356 (10)	0.20089 (9)	0.7295 (3)	0.0486 (8)	
O4	0.84766 (10)	0.11361 (10)	0.7265 (3)	0.0594 (9)	
C1	0.89801 (15)	0.07417 (16)	0.9562 (5)	0.0482 (13)	
C2	0.92849 (15)	0.08310 (14)	0.8356 (5)	0.0493 (13)	
H2	0.9575	0.0850	0.8605	0.059*	
C3	0.90355 (16)	0.04533 (15)	0.7341 (5)	0.0632 (15)	
H3A	0.8758	0.0454	0.7096	0.076*	
H3B	0.9228	0.0536	0.6574	0.076*	
C4	0.89048 (18)	−0.00300 (17)	0.7707 (6)	0.0700 (16)	
H4	0.8709	−0.0112	0.8479	0.084*	
C5	0.9313 (2)	−0.00798 (19)	0.8060 (7)	0.121 (3)	
H5A	0.9210	−0.0390	0.8318	0.181*	
H5B	0.9477	0.0123	0.8766	0.181*	
H5C	0.9512	−0.0004	0.7321	0.181*	
C6	0.8627 (2)	−0.03564 (18)	0.6648 (6)	0.109 (2)	
H6A	0.8541	−0.0663	0.6903	0.164*	
H6B	0.8805	−0.0279	0.5865	0.164*	
H6C	0.8356	−0.0337	0.6498	0.164*	
C7	0.97293 (15)	0.14987 (16)	0.7061 (4)	0.0469 (12)	
H7	0.9921	0.1377	0.6939	0.056*	
C8	0.98550 (14)	0.19273 (15)	0.6433 (4)	0.0399 (11)	
C9	0.96116 (15)	0.21653 (15)	0.6601 (4)	0.0415 (11)	
C10	0.97886 (17)	0.25974 (16)	0.6011 (5)	0.0570 (14)	
H10	0.9639	0.2763	0.6133	0.068*	
C11	1.01764 (19)	0.27800 (19)	0.5261 (5)	0.0657 (15)	
H11	1.0288	0.3068	0.4888	0.079*	
C12	1.04026 (17)	0.2539 (2)	0.5057 (5)	0.0657 (16)	
H12	1.0659	0.2659	0.4522	0.079*	
C13	1.02484 (15)	0.21226 (18)	0.5642 (5)	0.0563 (14)	
H13	1.0407	0.1965	0.5515	0.068*	
C14	0.80925 (14)	0.16091 (14)	0.8869 (4)	0.0444 (12)	
H14	0.7952	0.1409	0.8177	0.053*	
C15	0.78503 (15)	0.17820 (16)	0.9513 (5)	0.0534 (14)	
H15	0.7554	0.1698	0.9257	0.064*	
C16	0.80519 (17)	0.20759 (17)	1.0524 (5)	0.0536 (14)	
H16	0.7890	0.2187	1.0980	0.064*	
C17	0.85043 (16)	0.22109 (15)	1.0881 (4)	0.0421 (12)	
C18	0.87224 (13)	0.20153 (13)	1.0202 (4)	0.0332 (10)	
C19	0.91747 (13)	0.21233 (13)	1.0547 (4)	0.0329 (10)	
C20	0.94042 (15)	0.24385 (14)	1.1550 (4)	0.0409 (11)	
C21	0.98392 (16)	0.25152 (15)	1.1888 (5)	0.0515 (13)	
H21	1.0003	0.2719	1.2554	0.062*	
C22	1.00214 (15)	0.22934 (16)	1.1246 (5)	0.0491 (13)	
H22	1.0310	0.2342	1.1464	0.059*	
C23	0.97694 (15)	0.19928 (14)	1.0259 (4)	0.0430 (12)	
H23	0.9898	0.1842	0.9822	0.052*	
C24	0.87448 (19)	0.25339 (16)	1.1893 (5)	0.0551 (14)	
H24	0.8602	0.2667	1.2346	0.066*	
C25	0.91796 (18)	0.26461 (16)	1.2196 (4)	0.0565 (14)	
H25	0.9335	0.2864	1.2839	0.068*	

### Atomic displacement parameters (Å^2^)

**Table:**

	*U*^11^	*U*^22^	*U*^33^	*U*^12^	*U*^13^	*U*^23^
V1	0.0317 (5)	0.0396 (5)	0.0497 (5)	0.0173 (4)	−0.0018 (4)	−0.0068 (4)
N1	0.030 (2)	0.037 (2)	0.053 (3)	0.0164 (19)	0.0006 (19)	−0.0056 (19)
N2	0.029 (2)	0.036 (2)	0.045 (2)	0.0169 (18)	0.0024 (18)	0.0046 (19)
N3	0.027 (2)	0.033 (2)	0.045 (2)	0.0173 (18)	−0.0001 (18)	−0.0003 (17)
O1	0.0430 (19)	0.0417 (19)	0.062 (2)	0.0215 (17)	0.0091 (16)	0.0017 (16)
O2	0.070 (2)	0.064 (2)	0.068 (3)	0.035 (2)	0.0031 (19)	0.017 (2)
O3	0.047 (2)	0.0468 (19)	0.059 (2)	0.0287 (17)	0.0139 (17)	0.0082 (16)
O4	0.048 (2)	0.059 (2)	0.071 (2)	0.0265 (17)	−0.0164 (17)	−0.0237 (18)
C1	0.037 (3)	0.039 (3)	0.057 (4)	0.011 (3)	−0.005 (3)	−0.007 (3)
C2	0.036 (3)	0.035 (3)	0.077 (4)	0.018 (2)	−0.001 (3)	−0.002 (3)
C3	0.067 (4)	0.044 (3)	0.083 (4)	0.030 (3)	0.001 (3)	−0.005 (3)
C4	0.065 (4)	0.046 (3)	0.103 (5)	0.030 (3)	0.000 (3)	−0.005 (3)
C5	0.085 (5)	0.068 (4)	0.221 (8)	0.047 (4)	−0.010 (5)	0.009 (5)
C6	0.131 (6)	0.050 (4)	0.136 (6)	0.038 (4)	−0.034 (5)	−0.031 (4)
C7	0.041 (3)	0.051 (3)	0.059 (3)	0.031 (3)	−0.001 (3)	−0.013 (3)
C8	0.035 (3)	0.040 (3)	0.043 (3)	0.018 (2)	0.003 (2)	−0.001 (2)
C9	0.038 (3)	0.040 (3)	0.037 (3)	0.013 (3)	−0.010 (2)	−0.010 (2)
C10	0.070 (4)	0.048 (3)	0.052 (3)	0.028 (3)	−0.001 (3)	−0.001 (3)
C11	0.075 (4)	0.066 (4)	0.039 (3)	0.022 (4)	0.001 (3)	0.010 (3)
C12	0.046 (3)	0.086 (4)	0.043 (4)	0.017 (3)	0.001 (3)	0.006 (3)
C13	0.037 (3)	0.071 (4)	0.055 (3)	0.023 (3)	0.001 (3)	−0.007 (3)
C14	0.026 (3)	0.040 (3)	0.063 (3)	0.013 (2)	−0.005 (2)	0.001 (2)
C15	0.031 (3)	0.058 (3)	0.082 (4)	0.030 (3)	0.005 (3)	0.014 (3)
C16	0.054 (4)	0.063 (4)	0.061 (4)	0.042 (3)	0.023 (3)	0.022 (3)
C17	0.046 (3)	0.044 (3)	0.047 (3)	0.031 (3)	0.014 (3)	0.012 (2)
C18	0.033 (3)	0.029 (2)	0.038 (3)	0.015 (2)	0.007 (2)	0.010 (2)
C19	0.031 (3)	0.028 (2)	0.037 (3)	0.012 (2)	0.008 (2)	0.008 (2)
C20	0.046 (3)	0.039 (3)	0.035 (3)	0.019 (3)	0.006 (2)	0.002 (2)
C21	0.051 (3)	0.047 (3)	0.047 (3)	0.017 (3)	−0.009 (3)	−0.007 (2)
C22	0.029 (3)	0.057 (3)	0.059 (3)	0.020 (3)	−0.006 (3)	−0.001 (3)
C23	0.037 (3)	0.040 (3)	0.049 (3)	0.018 (2)	0.002 (2)	0.001 (3)
C24	0.071 (4)	0.047 (3)	0.057 (4)	0.036 (3)	0.023 (3)	0.005 (3)
C25	0.066 (4)	0.052 (3)	0.049 (3)	0.028 (3)	0.005 (3)	−0.010 (3)

### Geometric parameters (Å, º)

**Table:**

V1—O1	1.989 (3)	C7—H7	0.9300
V1—O3	1.941 (3)	C8—C13	1.407 (6)
V1—O4	1.587 (3)	C8—C9	1.414 (6)
V1—N1	2.042 (3)	C9—C10	1.405 (6)
V1—N2	2.125 (3)	C10—C11	1.369 (6)
V1—N3	2.340 (3)	C10—H10	0.9300
N1—C7	1.285 (5)	C11—C12	1.379 (6)
N1—C2	1.483 (5)	C11—H11	0.9300
N2—C14	1.333 (5)	C12—C13	1.367 (6)
N2—C18	1.369 (5)	C12—H12	0.9300
N3—C23	1.312 (5)	C13—H13	0.9300
N3—C19	1.344 (5)	C14—C15	1.385 (6)
O1—C1	1.290 (5)	C14—H14	0.9300
O2—C1	1.228 (5)	C15—C16	1.360 (6)
O3—C9	1.313 (5)	C15—H15	0.9300
C1—C2	1.541 (6)	C16—C17	1.403 (6)
C2—C3	1.531 (6)	C16—H16	0.9300
C2—H2	0.9800	C17—C18	1.394 (5)
C3—C4	1.506 (6)	C17—C24	1.429 (6)
C3—H3A	0.9700	C18—C19	1.423 (5)
C3—H3B	0.9700	C19—C20	1.403 (5)
C4—C6	1.497 (7)	C20—C21	1.398 (6)
C4—C5	1.511 (6)	C20—C25	1.425 (6)
C4—H4	0.9800	C21—C22	1.352 (6)
C5—H5A	0.9600	C21—H21	0.9300
C5—H5B	0.9600	C22—C23	1.384 (6)
C5—H5C	0.9600	C22—H22	0.9300
C6—H6A	0.9600	C23—H23	0.9300
C6—H6B	0.9600	C24—C25	1.353 (6)
C6—H6C	0.9600	C24—H24	0.9300
C7—C8	1.438 (6)	C25—H25	0.9300
			
O4—V1—O3	102.90 (15)	H6B—C6—H6C	109.5
O4—V1—O1	100.04 (14)	N1—C7—C8	125.1 (4)
O3—V1—O1	156.11 (12)	N1—C7—H7	117.5
O4—V1—N1	103.66 (14)	C8—C7—H7	117.5
O3—V1—N1	88.88 (13)	C13—C8—C9	118.9 (4)
O1—V1—N1	79.28 (13)	C13—C8—C7	117.7 (4)
O4—V1—N2	93.81 (14)	C9—C8—C7	123.4 (4)
O3—V1—N2	89.71 (12)	O3—C9—C10	118.4 (4)
O1—V1—N2	95.35 (12)	O3—C9—C8	123.7 (4)
N1—V1—N2	162.35 (14)	C10—C9—C8	118.0 (4)
O4—V1—N3	167.06 (14)	C11—C10—C9	121.6 (5)
O3—V1—N3	79.80 (12)	C11—C10—H10	119.2
O1—V1—N3	79.32 (11)	C9—C10—H10	119.2
N1—V1—N3	88.98 (12)	C10—C11—C12	120.3 (5)
N2—V1—N3	73.46 (13)	C10—C11—H11	119.8
C7—N1—C2	119.1 (4)	C12—C11—H11	119.8
C7—N1—V1	127.6 (3)	C13—C12—C11	119.9 (5)
C2—N1—V1	113.3 (3)	C13—C12—H12	120.1
C14—N2—C18	117.7 (4)	C11—C12—H12	120.1
C14—N2—V1	123.2 (3)	C12—C13—C8	121.3 (5)
C18—N2—V1	119.1 (3)	C12—C13—H13	119.3
C23—N3—C19	117.8 (4)	C8—C13—H13	119.3
C23—N3—V1	129.9 (3)	N2—C14—C15	123.2 (4)
C19—N3—V1	112.3 (3)	N2—C14—H14	118.4
C1—O1—V1	120.3 (3)	C15—C14—H14	118.4
C9—O3—V1	131.1 (3)	C16—C15—C14	119.1 (4)
O2—C1—O1	124.5 (5)	C16—C15—H15	120.4
O2—C1—C2	120.6 (5)	C14—C15—H15	120.4
O1—C1—C2	114.9 (4)	C15—C16—C17	120.1 (4)
N1—C2—C3	108.0 (4)	C15—C16—H16	119.9
N1—C2—C1	107.4 (4)	C17—C16—H16	119.9
C3—C2—C1	110.6 (4)	C18—C17—C16	117.3 (4)
N1—C2—H2	110.3	C18—C17—C24	119.5 (4)
C3—C2—H2	110.3	C16—C17—C24	123.2 (5)
C1—C2—H2	110.3	N2—C18—C17	122.5 (4)
C4—C3—C2	118.1 (4)	N2—C18—C19	117.2 (4)
C4—C3—H3A	107.8	C17—C18—C19	120.2 (4)
C2—C3—H3A	107.8	N3—C19—C20	122.8 (4)
C4—C3—H3B	107.8	N3—C19—C18	117.8 (4)
C2—C3—H3B	107.8	C20—C19—C18	119.4 (4)
H3A—C3—H3B	107.1	C21—C20—C19	116.9 (4)
C6—C4—C3	110.4 (5)	C21—C20—C25	123.9 (4)
C6—C4—C5	111.3 (5)	C19—C20—C25	119.1 (4)
C3—C4—C5	112.9 (4)	C22—C21—C20	120.0 (4)
C6—C4—H4	107.3	C22—C21—H21	120.0
C3—C4—H4	107.3	C20—C21—H21	120.0
C5—C4—H4	107.3	C21—C22—C23	118.7 (4)
C4—C5—H5A	109.5	C21—C22—H22	120.6
C4—C5—H5B	109.5	C23—C22—H22	120.6
H5A—C5—H5B	109.5	N3—C23—C22	123.8 (4)
C4—C5—H5C	109.5	N3—C23—H23	118.1
H5A—C5—H5C	109.5	C22—C23—H23	118.1
H5B—C5—H5C	109.5	C25—C24—C17	120.2 (4)
C4—C6—H6A	109.5	C25—C24—H24	119.9
C4—C6—H6B	109.5	C17—C24—H24	119.9
H6A—C6—H6B	109.5	C24—C25—C20	121.5 (4)
C4—C6—H6C	109.5	C24—C25—H25	119.3
H6A—C6—H6C	109.5	C20—C25—H25	119.3
			
C1—C2—C3—C4	−62.4 (6)		

### Hydrogen-bond geometry (Å, º)

**Table:**

*D*—H···*A*	*D*—H	H···*A*	*D*···*A*	*D*—H···*A*
C12—H12···O4^i^	0.93	2.44	3.311 (6)	156
C24—H24···O1^ii^	0.93	2.51	3.224 (6)	134

Symmetry codes: (i) *y*+1, −*x*+*y*+1, −*z*+1; (ii) −*x*+5/3, −*y*+1/3, −*z*+7/3.
